# An Unusual Uterine Extension Revealing a Congenital Diverticulum: Insight Into Müllerian Duct Development

**DOI:** 10.7759/cureus.111246

**Published:** 2026-06-21

**Authors:** Nidhi Sunhare, Padamjeet Panchal

**Affiliations:** 1 Anatomy, All India Institute of Medical Sciences, Patna, Patna, IND

**Keywords:** congenital malformation, embryological fusion defect, endometrial cavity, genitourinary anomaly, müllerian duct anomaly, myometrium, uterine diverticulum

## Abstract

The genitourinary system undergoes a complex development, making it prone to congenital anomalies. Müllerian duct anomalies are among these, affecting the uterus, cervix, and vagina due to errors in ductal fusion or septal resorption during development. During cadaveric dissection of a 55-year-old female, a 4.6 cm tubular fibrous extension was identified, projecting from the uterine fundus to the posterior aspect of the anterior abdominal wall near the umbilicus. The uterus itself was poorly developed, with an atrophied body, cervix, and conical fundus. A thick fibrous band connected the fundus to the inner abdominal surface. Histological examination (hematoxylin and eosin staining) revealed an atrophied myometrial wall, a poorly developed cystic endometrial cavity with cystic glands and papillary fronds, and fibromuscular tissue covered by serosa. Bilateral adnexal nodules showed fibrocollagenous tissue with cavernous vascular spaces but no ovarian tissue. No cilia, inflammation, endometriosis, dysplasia, or malignancy were identified. Wolffian remnants were excluded based on myometrial and endothelial composition. The findings are consistent with a true uterine diverticulum, a rare Müllerian duct fusion anomaly. Incomplete midline fusion likely created a focal wall defect that dilated under intrauterine pressure. Clinically, such anomalies may predispose to ectopic pregnancy, abnormal uterine bleeding, or dysmenorrhea, and pose surgical risks if unrecognised during cesarean sections or myomectomies.

## Introduction

The genitourinary system develops through a series of complex, highly coordinated embryological processes, making it one of the most anomaly-prone systems in the human body [1]. Developmental abnormalities of the genitourinary tract account for 14-40% of congenital anomalies detected on prenatal sonography, emphasising their clinical significance in both obstetric and surgical practice [1,2].

Among these anomalies, Müllerian duct anomalies (MDAs) represent a well-recognised but incompletely understood spectrum of congenital malformations affecting the female internal genitalia. The Müllerian (paramesonephric) ducts are the primordial structures that, under normal developmental conditions, give rise to the fallopian tubes, uterus, cervix, and upper vagina [3,4]. Their normal development depends on the successful completion of three sequential phases: organogenesis, midline ductal fusion, and septal resorption. Disruption at any of these stages can result in a wide spectrum of structural anomalies ranging from uterine agenesis to a septate uterus, each carrying distinct reproductive and clinical implications [3,5].

While the more common MDAs - such as bicornuate, septate, and didelphys uteri - are well-documented in the literature and classified under the American Society of Reproductive Medicine (ASRM) 2021, the European Society of Human Reproduction and Embryology (ESHRE), and the European Society for Gynaecological Endoscopy (ESGE) 2013 classification systems. The ASRM classification was modified in 2021 to standardise terminology, ease identification in scientific databases, educate and facilitate use by providers, and promote patient awareness. This modification removed the previous class-based system and instead describes anomalies involving the uterus, cervix, and vagina, dividing them into nine descriptive categories. These categories are Müllerian agenesis, cervical agenesis, unicornuate uterus, uterus didelphys, bicornuate uterus, septate uterus, longitudinal vaginal septum (LVS), transverse vaginal septum, and complex anomalies [4]. Certain anomalies remain exceptionally rare and poorly characterised. Among these, true uterine diverticula are among the least frequently reported entities. While the terminology for uterine diverticulum varies across published classifications, it is most commonly described as a pouch-like outpouching of the uterine wall that communicates with the endometrial cavity and is composed of tissues homologous to the parent uterine tissue [6,7]. Unlike pregnancy-associated sacculations, which are transient and functionally driven, true congenital uterine diverticula are hypothesised to arise from a localised failure of midline Müllerian duct fusion. This mechanism remains theoretical - inferred from the anatomical and histological features of reported cases rather than from direct embryological evidence - whereby a structurally vulnerable midline area dilates under uterine stress to form a diverticulum [2,8].

The clinical presentation of uterine diverticula is variable and often non-specific, encompassing dysmenorrhoea, abnormal uterine bleeding, infertility, and in some cases, ectopic pregnancy [9,10]. Their rarity and tendency to mimic other pelvic pathologies, such as degenerated leiomyomas, ovarian cysts, or accessory uterine masses, further complicate timely diagnosis [7,11]. While the majority of reported cases involve the uterine cervix or lower uterine segment, extensions from the uterine fundus to the anterior abdominal wall represent an exceptionally unusual variant, with only a handful of cases documented globally [2,12].

Cadaveric dissection has historically served as an invaluable tool for the discovery and characterisation of rare anatomical anomalies that may remain clinically silent throughout life. The accidental identification of an unusual uterine extension during routine dissection provides a rare opportunity to study its structural and histological features in the absence of clinical symptoms or surgical intervention.

## Case presentation

**Figure 1:**
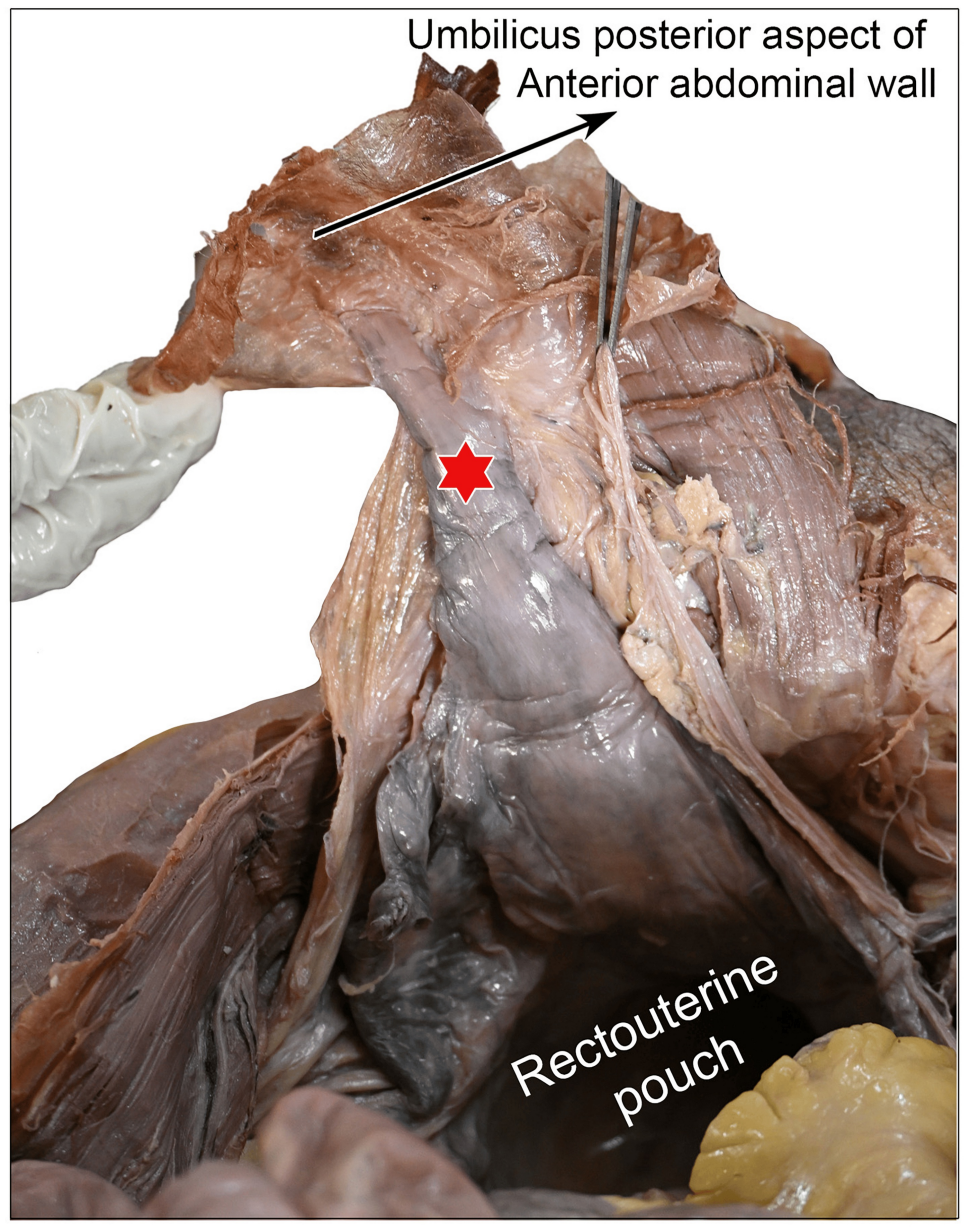
Cadaveric dissection of the female pelvis and anterior abdominal wall (posterior view) demonstrating an adherent, conical, tapered, elongated extension arising from the fundus of the uterus and extending toward the umbilicus and the rectouterine pouch. The red star (★) marks the conical, tapered, elongated extension arising from the fundus of the uterus.

A 55-year-old female embalmed cadaver was dissected and examined during routine undergraduate dissection sessions in the dissection hall. During the dissection of the pelvic region, an unusual anatomical finding was noted. On gross examination, the uterus appeared atrophied, displaying a reduced body and cervix with a conical fundus, consistent with the involutional changes expected in a postmenopausal uterus. A distinct tubular fibrous extension was identified projecting from the external surface of the uterine fundus, extending anteriorly toward the inner surface of the infraumbilical anterior abdominal wall. The total length of this uterine extension measured 4.6 cm. The structure had a broader base at its attachment to the uterine fundus and tapered distally, becoming more tubular as it approached the anterior abdominal wall. No accompanying vessels, nerves, or accessory structures were identified in association with this extension. The terminal part of the tubular extension became a thicker fibrous band and was adherent to the inner surface of the anterior abdominal wall. While searching for the ovaries, bilateral nodular tissues were noted in the parametrial/adnexal region, lateral to the uterus and below the fallopian tubes; however, these did not demonstrate any identifiable ovarian tissue on gross examination (Figure 1).

Photographic documentation of the specimen was performed in situ prior to dissection. The anomalous conical segment was meticulously dissected and detached from the anterior abdominal wall. The specimen was examined in cross-section, revealing a slit-like small endometrial cavity within the uterus. This cavity extends into the diverticulum, but in the distal part, it becomes rudimentary, narrow, and tubular and is later obliterated. It is plausible that the narrowing or occlusion of the distal portion of the extension may have developed over time. The uterine wall was notably thickened at the broader base of the diverticulum, and progressively thinned as it was traced distally. Subsequently, tissue sections were taken from different areas of the uterus for histological analysis.

**Figure 2:**
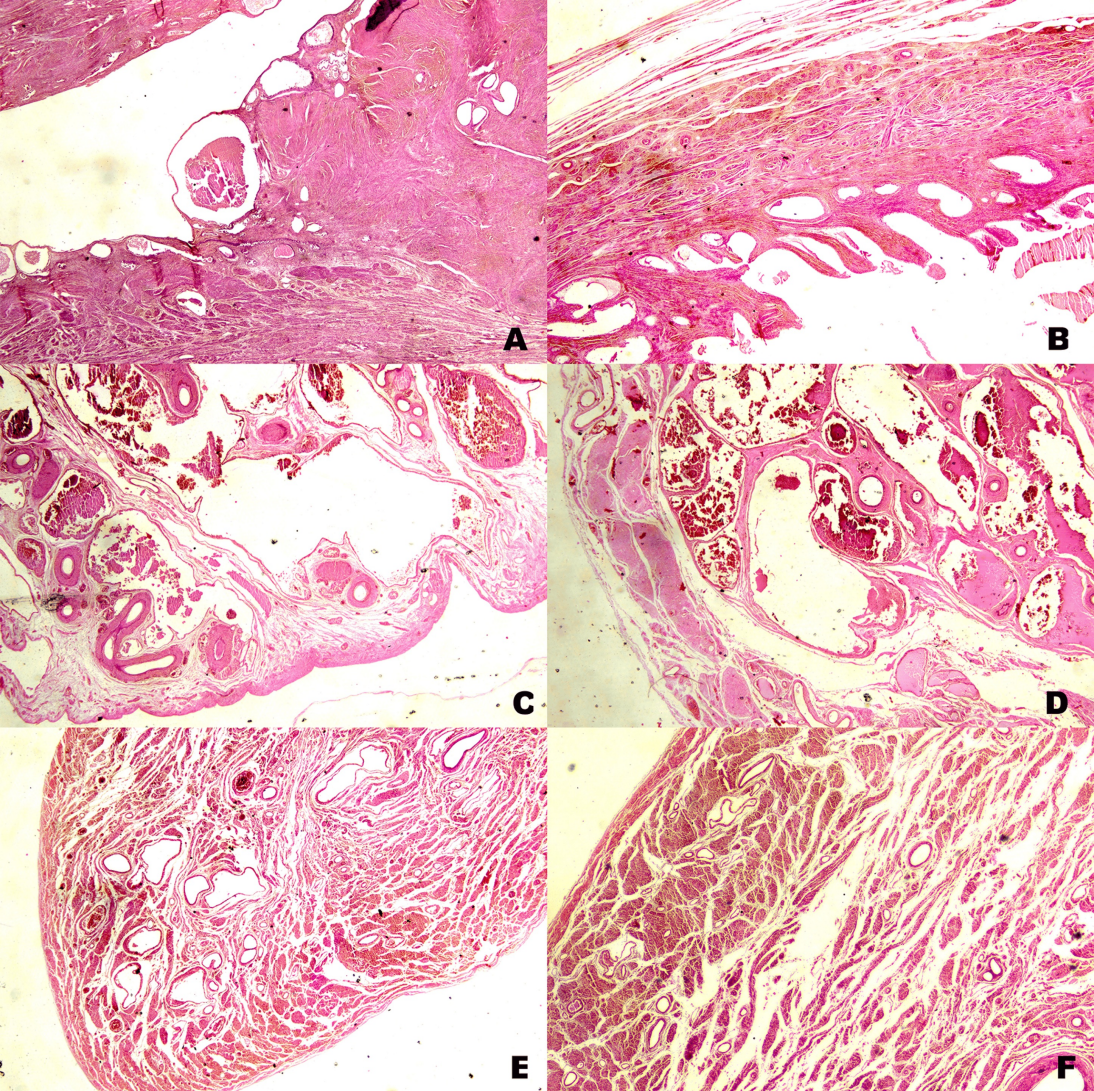
Histological sections taken from the uterine wall stained using the standardised hematoxylin and eosin technique. Slides A and B: magnification of about 20x - revealing a muscular atretic wall of the myometrium and a poorly developed cystic endometrial cavity, comprising of cystic endometrial glands and papillary fronds on the endometrial surface. Slides C and D: magnification of about 20x - sections from the nodular tissues on the bilateral adnexal aspects did not reveal any ovarian tissue; instead, it showed a nodular fibrocollagenous tissue with cavernous vascular spaces throughout. Slides E and F: magnification of about 20x - the band-like fibrous tissue connecting the uterus with the abdominal wall also demonstrated a fibromuscular tissue covered with a serosa. Histological sections courtesy of Dr. Shreekant Bharti, Additional Professor, Department of Pathology, AIIMS Patna.

The tissue sections were subsequently embedded in paraffin in accordance with established laboratory protocols. Paraffin blocks were sectioned to a thickness of 10 μm and stained using the standardised hematoxylin and eosin (H&E) technique. Microscopic examination was performed using a light microscope across a range of magnifications. Histological examination revealed an atrophied myometrial wall with a poorly developed cystic endometrial cavity. Cystic endometrial glands and papillary fronds were identified on the endometrial surface. The bilateral adnexal nodular tissues demonstrated fibrocollagenous tissue with cavernous vascular spaces, with no evidence of ovarian tissue. The fibrous band connecting the uterus to the anterior abdominal wall was composed of fibromuscular tissue covered by a serosa. The tissue was characterised by dense smooth muscle, prominent vascularity, and uterine-like architecture with partial luminal continuity, consistent with myometrial and endometrial composition. The histological findings confirmed the exclusion of Wolffian remnants (Figure 2).

Importantly, no evidence of inflammation, endometriosis, dysplasia, or malignancy was identified in any of the sections examined. The overall histological profile of the extension - including its smooth muscle composition, vascularity, and uterine-like architectural organisation - was consistent with a true uterine diverticulum of congenital Müllerian duct origin.

## Discussion

The present case describes an unusual tubular uterine extension, incidentally identified during cadaveric dissection, projecting from the uterine fundus to the anterior abdominal wall. Based on gross anatomical observation and histological analysis, the findings are consistent with a congenital uterine diverticulum - a rare Müllerian duct fusion anomaly with fewer cases documented in the world literature [6,7].

Embryological basis

The Müllerian (paramesonephric) ducts are the embryological precursors of the female internal genitalia, giving rise to the fallopian tubes, uterus, cervix, and upper vagina through three sequential developmental phases: organogenesis, midline fusion, and septal resorption [3,4]. Normal development of the uterus requires the successful fusion of the paired Müllerian ducts in a caudal-to-cranial direction, followed by resorption of the intervening septum to form a single unified uterine cavity [5]. Disruption at the fusion phase creates a spectrum of anomalies ranging from complete non-fusion, as seen in uterus didelphys, to partial or localised fusion defects that may manifest as uterine diverticula [3].

In the present case, the attachment of the extension to the uterine fundus, combined with its histological resemblance to uterine tissue, strongly supports an embryological connection to the Müllerian ducts. It is hypothesised that incomplete midline fusion of the Müllerian ducts created a localised area of structural weakness at the fundus. This weakened zone, subjected to intrauterine pressure over time - particularly during menstruation or possible prior pregnancies - may have progressively expanded into the observed tubular diverticular extension [2,7]. This hypothesis is consistent with previously proposed mechanisms for uterine diverticulum formation, wherein a focal midline fusion defect creates a vulnerable site in the uterine wall that gradually dilates under physiological stress [2,8].

Histological findings and confirmation

Histological examination played a central role in confirming the nature of this extension. The identification of dense smooth muscle, prominent vascularity, and uterine-like architecture with partial luminal continuity closely mirrors the histological composition of the normal uterine wall [2]. These findings are consistent with previously established criteria for a true uterine diverticulum, which require the presence of smooth muscle in the wall, endometrial glands, a connection to the uterine cavity via a tract, and coverage by a peritoneal serosa [6,10].

The absence of cilia on histological examination is a notable finding, as it effectively excludes the possibility of an accessory fallopian tube, which would be expected to demonstrate ciliated epithelium [2]. Furthermore, the myometrial and endometrial composition of the extension ruled out Wolffian (mesonephric) remnants, which typically present as fibrocollagenous structures without smooth muscle or uterine-like organisation [9]. The bilateral adnexal nodular tissues, showing fibrocollagenous tissue with cavernous vascular spaces in the absence of ovarian tissue, are consistent with previously reported accessory adnexal structures associated with Müllerian anomalies [13].

The cystic endometrial glands and papillary fronds identified within the poorly developed endometrial cavity further support the Müllerian origin of this structure, as these features are characteristic of functional endometrial tissue. The atrophied nature of the myometrial wall and endometrial cavity is consistent with the post-menopausal status expected in a 55-year-old cadaver, in whom hormonal withdrawal would lead to progressive endometrial atrophy [10].

Comparison with existing literature

The present case shares notable similarities with the case documented by Sagoo et al. [2], who reported a tube-like extension with a clear base attached to the fundus of a 96-year-old female cadaver, whose tapering distal end was attached to the inner surface of the infraumbilical anterior abdominal wall. Both cases demonstrated a uterine-like histological composition, the absence of cilia, and attachment to the anterior abdominal wall, thereby reinforcing the classification of such extensions as true uterine diverticula of congenital origin. A comparable case was also reported by Schauffler, who described a menstruating tract extending from the uterus to the anterior abdominal wall, histologically composed of smooth muscle bundles, connective tissue, vasculature, and endometrial glands, further supporting the Müllerian origin of such structures [12].

Primary uterine diverticula have been reported to arise most commonly from the cervix, accounting for approximately 59% of documented cases, with the remaining arising from the uterine corpus [10]. The fundal location observed in the present case, with extension to the anterior abdominal wall, represents an exceptionally rare variant, highlighting the diverse anatomical manifestations of Müllerian fusion defects. Noguchi et al. [11] proposed that primary uterine diverticula arise from unilateral distal non-fusion of the Müllerian ducts, a hypothesis that aligns with the localised fundal defect observed in the present case.

Unlike secondary uterine diverticula, which arise as a consequence of uterine surgery such as caesarean section or myomectomy and are characterised by thin myometrial walls and a history of iatrogenic injury [14,15], the present case demonstrates no evidence of surgical scarring or fibrosis at either end of the extension. The absence of inflammation and the well-organised fibromuscular architecture further support the congenital rather than acquired nature of this diverticulum.

Several conditions must be considered in the differential diagnosis of an anomalous uterine extension. Accessory uterine tubes, while sharing a similar tubular morphology, can be excluded in the present case by the absence of cilia on histological examination and the lack of fimbriated ends [2]. Abdominal wall endometriosis, which may present as a soft tissue extension involving the anterior abdominal wall, was excluded by the absence of endometriotic glands within the abdominal wall tissue and the lack of inflammatory changes [16]. Cavitated accessory uterine masses, as described by Acién et al. [13], typically present in young women with severe dysmenorrhoea and are located at the level of the round ligament insertion, features not consistent with the present case. Secondary or pregnancy-associated uterine sacculations, characterised by thin myometrial walls and a history of prior intrauterine pregnancy, are also inconsistent with the histological findings of this case [6].

Clinical implications

Although this diverticulum was identified incidentally in a cadaveric specimen, the clinical implications of such an anomaly are significant if present in a living patient. Uterine diverticula have been associated with a range of gynaecological complications, including abnormal uterine bleeding, dysmenorrhoea, infertility, and ectopic pregnancy [9,10]. Ectopic pregnancies occurring in the fundal region carry an approximately 70% likelihood of rupture and may be misdiagnosed as abdominal pregnancies [9]. It has been hypothesised in the literature that, in the context of assisted reproductive technology, an undetected diverticulum may impair embryo transfer and contribute to implantation failure by acting as a reservoir that obstructs access to the endometrial cavity or misdirects the transfer catheter [10,17]. This remains a speculative, case-derived mechanism rather than an established cause.

From a surgical perspective, an unrecognised diverticulum extending to the anterior abdominal wall poses significant risks during procedures involving abdominal incisions, such as caesarean sections, laparotomies, or laparoscopic interventions. Inadvertent incision into such a structure could result in uterine perforation, haemorrhage, or poor wound healing [2]. Preoperative imaging, particularly MRI, plays a crucial role in the detection and characterisation of such anomalies, given its superior soft tissue resolution and ability to delineate both internal and external uterine anatomy [3,4].

Limitations

This study is subject to certain limitations inherent to cadaveric case reports. The absence of a detailed clinical history limits the ability to correlate the anatomical findings with potential symptoms experienced during the individual's lifetime. Furthermore, the effects of long-term formalin fixation on tissue morphology may have introduced some degree of histological artefact, potentially obscuring finer structural details. The small sample size, comprising a single cadaveric case, precludes broader generalisations regarding the prevalence or clinical behaviour of this anomaly.

## Conclusions

The present case enriches the existing body of anatomical and clinical literature on true congenital uterine diverticula. The combination of gross anatomical observation and histological analysis provides compelling evidence for the classification of this extension as a Müllerian duct fusion anomaly. Increased awareness of this rare entity among anatomists, gynaecologists, and surgeons is essential to ensure accurate diagnosis, appropriate surgical planning, and optimal patient management when such anomalies are encountered in clinical practice.
